# An *In silico* Approach to Identify High Affinity Small Molecule Targeting m-TOR Inhibitors for the Clinical Treatment of Breast Cancer

**DOI:** 10.31557/APJCP.2019.20.4.1229

**Published:** 2019

**Authors:** Khushboo Patidar, Umesh Panwar, Sugunakar Vuree, Jajoriya Sweta, Manpreet Kaur Sandhu, Anuraj Nayarisseri, Sanjeev Kumar Singh

**Affiliations:** 1 *In silico Research Laboratory, Eminent Biosciences, Madhya Pradesh, *; 4 *Bioinformatics Research Laboratory, LeGene Biosciences Pvt Ltd., Indore, *; 2 *Computer Aided Drug Designing and Molecular Modeling Lab, Department of Bioinformatics, Alagappa University, Karaikudi, Tamil Nadu,*; 3 *Department of Biotechnology, Lovely Faculty of Technology and Sciences, Division of Research and Development, Lovely Professional University, Phagwara, Punjab, India. *

**Keywords:** Breast cancer, mTOR, Schrodinger Suite, MM-GBSA, Molecular Docking, virtual screening

## Abstract

Breast cancer is the most frequent malignancy among women. It is a heterogeneous disease with different subtypes defined by its hormone receptor. A hormone receptor is mainly concerned with the progression of the PI3K/AKT/mTOR pathway which is often dysregulated in breast cancer. This is a major signaling pathway that controls the activities such as cell growth, cell division, and cell proliferation. The present study aims to suppress mTOR protein by its various inhibitors and to select one with the highest binding affinity to the receptor protein. Out of 40 inhibitors of mTOR against breast cancer, SF1126 was identified to have the best docking score of -8.705, using Schrodinger Suite which was further subjected for high throughput screening to obtain best similar compound using Lipinski’s filters. The compound obtained after virtual screening, ID: ZINC85569445 is seen to have the highest affinity with the target protein mTOR. The same result based on the binding free energy analysis using MM-GBSA showed that the compound ZINC85569445 to have the the highest binding free energy. The next study of interaction between the ligand and receptor protein with the pharmacophore mapping showed the best conjugates, and the ZINC85569445 can be further studied for future benefits of treatment of breast cancer.

## Introduction

*Breast cancer*

Breast cancer is the prevalent malignancy among women worldwide (Cidadoet al., 2012). It often described as a heterogeneous disease with different subtypes, defined by its hormone receptor. All the current treatment decision for advanced breast cancer based on these biomarkers (Chan et al., 2005). Despite of the advancement in detection and targeted therapies, the mortality rate remains high because of the development of resistant cell lines. Thus, metastasis breast carcinoma remains an ineradicable disease by modern remedial approaches. So, a better perspective must be needed to thrive new treatment regimen.

According to the American cancer society, around 232,340 recent cases of invasive breast cancer and 39,620 breast cancer deaths are anticipated to happen among US women in 2013 (DeSantis et al., 2014). Over the past two decades, breast cancer occurrence and mortality rates have been increasing rapidly. Breast cancer alone constitutes between 25% and 15% of all cancer cases and cancer deaths among women respectively (Torre et al., 2015). Developed countries comprise the number of breast cancer cases and deaths, incidence and mortality rate manifest the availability of the detection and treatment procedure. So, prolonged studies and efforts are required to provide better and easy detection and treatment procedures to all kinds of the population (DeSantis et al., 2014; Torre et al., 2015).

Breast cancer is a complicated and multifactorial disease. Therefore, so many risk factors associated with breast cancer, such as age, body mass index, family history having abnormal BRCA1 or BRCA2 gene, hormonal factors like- menarche at an early age, menopause at a late age, late pregnancy, little or no breastfeeding, oral contraceptive, and hormone replacement therapy (Laamiri et al., 2016). The precise interpretation of predisposing factors might be helpful to the development of the novel treatment to prevent breast cancer. The top-most encouraging efforts require understanding the gene, gene-environment, or gene-gene interaction.

The leading cause of cancer is the genetic mutation which occurs as a result of genetic instability and environmental factors. In most of the breast cancer, genetic alteration arises during an individual’s lifetime and are present only in specific cells of the breast. These types of changes are called somatic mutation and are not inherited. A different kind of genetic changes, which is classified as germline mutation, are typically inherited from parent to their offspring.

Mutation in the number of genes is involved in causing breast cancer. Regarding high-risk family history, the most important genes are BRCA1 and BRCA2 (Ford et al., 1998). Variation in these two genes can increase the chance of developing the tumors by 80%. Therefore, early recognition of carrier among affected women is pivotal to suggest the specialist to determine the best suitable treatment plan (D’Argenio et al., 2015). 

*mTOR pathway*

mTOR kinase is an intracellular signaling pathway which controls the various actions of our body such as cell division, cell survival, cellular morphology, protein synthesis and integration of metabolism. mTOR kinase signaling is activated in multiple cancer, guided by variation in the gene-coding receptor tyrosine kinase, Ras, PI3K, and PTEN that is involved in numerous cellular processes (Bhagwat et al., 2011). This protein is a component of two multi-subunit complexes known as mTOR1 and mTOR2. The mTOR1 complex is sensitive to anticancer drug Rapamycin, an allosteric inhibitor of mTORC1 results in complete disruption of the complex. The mTOR2 complex is considered as rapamycin-insensitive, regulates actin cytoskeleton by phosphorylating the survival kinase AKT at SER 473. mTOR is mainly activated by some intracellular and extracellular signals such as growth factors, nutrient status, energy metabolism and Oxygen level (O’Reganet al., 2011; Aarthy et al., 2017).

*Dysregulation of the mTOR pathway*

The aberrant activation of mTOR pathway identified in most of the cancer including breast cancer and its hyperactivation commonly associated with cell growth, cellular proliferation and neogenesis (García-Echeverría et al., 2010). For instance, in the variety of cancer phosphatase and tensin homologue deleted in chromosome 10(PTEN) is mutationally inactivated, leads to an increase in mTOR activity. This hyperactivated mTOR gene results in the production of mRNAs that encode growth factors, cell death inhibitor, angiogenesis factors, cell growth inducer which overall promote carcinogenesis. Therefore, mTOR must be specifically targeted as an anticancer therapy for the treatment of cancer (Dowling et al., 2007). Overall features of the mTOR signaling pathway have provided a higher level of interest in targeting mTOR as a potential therapeutic agent for effective treatment (Xie et al., 2016).

## Materials and Methods

*Methodology*

*System Configuration*

The present study was performed using a Schrödinger’s Drug Discovery Suite 2015 on the platform of Cent OS Linux 6.5 version. Molecular Dynamics simulations were carried out using an academic version of Desmond 2015.

*Selection of Protein Target and known inhibitors*

The three-dimensional structure of the target protein mTOR was retrieved from Protein Data Bank – PDB (www.rcsb.org) with PDB ID: 5H64. Established inhibitors for mammalian target of rapamycin (mTOR) were explored using NCBI’s PubChem compound database (Chandrakar, B. et al., 2013; Rao DM. et al., 2010). The total of 40 compounds were selected to consider the best-anchored compound. The selected inhibitors along with their PubChem ID are shown in Table 1.

*Preparation of Protein*

Targeted three-dimensional structural coordinates was pre-processed using Protein Preparation Wizard module in Schrödinger Suite (Protein preparation wizard, Schrodinger, 2017) (Sharda et al., 2017; Bandaru et al., 2017) by implying the parameters like assigning bond orders, zero-order bonds to metal atoms, selenomethionine to methionine conversion, filling absent hydrogen’s, capping termini, side chains and loops, and removing waters beyond 5 Å distance surrounding the co-crystallized ligand (Bandaru et al., 2017; Shameer et al., 2017; Nasr et al., 2015; Khandekar et al., 2016; Singh et al., 2019). Further, tautomerization and protonation states were predicted in favor of ligand at pH 7.00. Lastly, the protein hydrogen bonds were optimized to renovation the overlying hydrogens and minimized using OPLS-2005 force field with root mean square deviation (RMSD) value of 0.30 A° (Jorgensenet al., 1996; Glide, 2015; Reddyet al., 2014; Patidar et al., 2016; Shaheen et al., 2015; Praseetha et al., 2016; Babitha et al., 2015).

*Lead Compounds and Database preparation*

All the 40 compounds retrieved from NCBI’s PubChem Database and a large chemical library of drug-like compounds – Zinc databases were prepared using the Ligand preparation module (LigPrep, 2017)(Dunna et al., 2015; Dunna et al., 2015; Bandaru et al., 2015) of molecular modeling package with suitable parameters like optimization, ring conformation, 2D to 3D conversion, determination of protomers, tautomers, and ionization states at pH 7.0, along with partial atomic charges using OPLS_2005 force field (Bandaruet al., 2014; Jorgensen et al., 1996; LigPrep, 2015; Sinha et al., 2015).

*Active Site Prediction and Receptor Grid Generation*

Using SiteMap module in Schrodinger Suite, Ligand – Binding site of the receptor was predicted. Based on top ranked site score, five potential active sites were analyzed. Site2 was taken for docking analysis based on highest site score along with presence of hydrophobic and charged amino acids (Sinha et al., 2014; Reddy et al., 2014; Panwar et al., 2017).Using Receptor Grid Generation module in Schrodinger, the grid generation was performed on prepared protein with the help of site score from SiteMap (Bandaru et al., 2014; Bandaru et al., 2013). The atoms of protein were fixed within the default parameters of the radii of Vander Waal’s scaling factor of 1 Å with partial charge cut-off of 0.25Å using OPLS_2005 force field (Vuree et al., 2013).

*Molecular Docking*

Firstly, all the selected 40 compounds were docked into the generated grid of prepared protein using Glide XP (extra precision) module, Schrodinger with default parameters. Glide score is used to rank the various poses of ligand in complex with receptor, where the higher negative values reveal strong binding interaction of protein-ligand. Generally, Glide score is calculated based on the equation:

Glide score = 0.065 * vdW + 0.130 * Coul + Lipo + Hbond + Metal + BuryP +RotB + Site (1)

Where,vdW =vanderWaalsenergy, Coul=Coulomb energy, Lipo=Hydrophobicinteractions, 

H-bond=Hydrogenbonds, 

Metal=Metalbindingterms,

BuryP=Penaltyfor buried polargroups,

RotB=Freezingrotatablebonds, Site=Polar interaction inthebindingsite.

Based on the glide docking score the best-docked complex was selected for further studies of virtual screening (Suryanarayanan et al., 2005; Singh et al., 2016).

*High throughput Virtual Screening*

Virtual screening is one of the highly advanced computing processes to identify the novel drug candidate from large chemical libraries against biological target. Herein, the trio of HTVS, SP, and XP from Glide, Schrodinger was utilized to filter the best configuration of ligand with highest docking score in every step of virtual screening trio (Aarthyet al., 2017; Suryanarayanan et al., 2005; Singh et al., 2016). Finally, the best ranking compounds were selected using criteria of docking score for further studies of ADMEprediction. 

*Pharmacophore studies*

Pharmacophore studies involve different types of interactions between ligand and receptor. This study includes H-bond interaction, electrostatic interaction, hydrophobic interaction, and aromatic interaction done by using Accelrys Discovery Studio 3.5 DS Visualizer (Visualizer, Accelrys Inc, 2012; Basak et al., 2016; González-Díaz et al., 2016; Kelotra et al., 2014; Kelotra, et al., 2014; Majhi et al., 2018; Khandelwal et al., 2018; Sharma et al., 2018; Sinha et al., 2018).

*ADME and Toxicityprofile*

As per olden research, many of drugs have been failed during clinical studies due to poor ADME/T; it leads to high cost of loss to pharmaceutical companies. Thus, QikProp, Schrodinger was taken forward for profiling the ADME properties of selected hits from screening. It evaluates the drug likeness and pharmaceutical properties such as molecular weight, aqueous solubility (Q^P^log^S^), octanol/water (log^P^), brain/blood partition coefficient (Q^P^log^BB^), CNS, hydrogen bond donors and acceptors along with Lipinski rule of five and Jorgensen rule of three. Following to these, the Human intestinal absorption (HIA), Blood brain barrier (BBB), AMES toxicity and LD50 were also generated to check compounds toxicity effect using online web server tool admetSAR (QikProp 2015; Cheng et al., 2012).

*MM-GBSA (Molecular Mechanics, the Generalized Born model and Solvent Accessibility) – free binding energy calculation*

MM–GBSA, an efficient computational approach in Prime module of Schrodinger suit 2015 (Schrodinger, Inc., LLC, New York, USA), is worthwhile to calculate the relative binding free energy and to improve docking score after docking analysis from bio-molecular system. The binding free energy determines by ΔG_bind_, represented by following equation (Prime, Schrodinger, 2015; Lyne et al., 2006):

ΔG_bind_ = ΔE + ΔG_solv_ + ΔG_SA_

Where, ΔG_bind_ = Binding Free Energy, ΔE = Difference of energy minimization between receptorligand complex & the energies of receptor and ligand Where, ΔE = E_complex_ – E_receptor _– E_ligand_, ΔG_solv_ = Difference of electrostatic solvation energy of the receptor-ligand complex & the energies of receptor and ligand Where, ΔG_solv_ = G_solv(complex)_ – G_solv (receptor)_ – G_solv (ligand)_, ΔG_SA_ = Difference of Surface area energies of the receptor-ligand complex and the energies of receptor and ligand:

Where, ΔG_SA_ = G_SA(complex)_ – G_SA_ _(receptor)_ – G_SA_ _(ligand)_

The Prime module applies a surface generalized Born model which makes use of a Gaussian surface for enhanced demonstration of a solvent accessible surface area (Suryanarayanan et al., 2005; Singh et al., 2016; Prime, Schrodinger, 2015; Lyne et al., 2006).

*BOILED-Egg plot*

A Brain or intestinal EstimateD permeation method (BOILED-Egg) is a predictive model which predicts the bioavailability of the drugs over gastrointestinal absorption and brain penetration. There are five parameters which defines the Cartesian coordinates of both ellipses includes MW, TPSA, MLOGP, GI and BBB. Classification of Egg-plot include the yolk (physiochemical space for BBB permeation), the white (physiochemical space for HIA absorption), and the outside grey region stands for the molecule which predicted low absorption and limited brain penetration properties. The molecule which are placed in grey region are counted as remarked (Padmini et al., 2019; Divya et al., 2019; Palak et al., 2019, Trishang et al., 2019). 

To proceed for further analysis of Egg-plot, compound ID of the top three best drug from each of established docked and virtual screened docked was retrieved. Observable result were analyzed based on the different parameters used for Egg-plot.

## Results

Herein this study, a highly significant virtual screening process was applied to find out an effective leading compound using Schrodinger Software. 

*Docking and virtual screening results*

Molecular docking of top 40 known inhibitors was identified with great binding interaction within the same active site. Later, SF1126, the best one protein-ligand complexes were analyzed as perfect binding confirmation with better docking score, XP Score, glide energy, and Glide emodel, tabulated (Table 2) which were taken forward for virtual screening process against Zinc NCD database. The resulted top 10 compounds with appropriate pharmacological including drug like properties as potent inhibitors against targeted protein with highest docking score, glide score and glide energy, shown (Table 3). The resulted compound ID:ZINC85569445shows the best affinity with the target protein with docking score of -10.607 kcal/mol. The binding mode analyses of the ZINC85569445 were described in detailed.

*Binding mode of Compound ZINC85569445 with the receptor*

**Figure 1:**
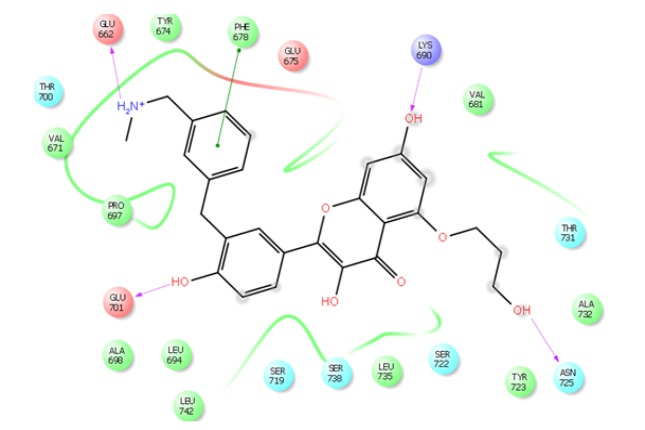
Compound ID:ZINC85569445 Shows High Affinity with mTOR Protein

The ligand ZINC85569445was identified with highest docking score -10.607 kcal/mol, glide energy -61.060 kcal/mol, glide Emodel -82.947 kJ/mol. Hydrogen bond interactions were identified with the amino acids Glu662, Lys690, Glu701, Asn725; in which, amine group of compound interacted with oxygen of Glu662 with a distance of 2.01 Å, three carboxyl group of compound interacted respectively with amino group of Lys690 with a distance of 2.43 Å, oxygen of Glu701 with a distance 1.96 Å, and oxygen of Asn725 with a distance 2.07 Å. Amino acids residues Val671, Tyr674, Phe678, Val681, Leu694, Pro697, Ala698, Tyr723, Ala732, Leu735, Leu742 were observed as hydrophobic residues. The 2D profile interaction diagram was represented in Figure 1.

*Pharmacophore Studies*

Pharmacophore mapping helps to understand the interaction between ligand and receptor molecule. In the active site of target protein, compound ID:ZINC85569445 shows considerable interaction. It shows electrostatic interaction with Ser722 and Lys690, while the H-bond was observed with Glu662, Lys690, Glu701, and Asn725. Figure 4, depicts the aromatic interaction where Phe678, Thr731 actively participated. 

**Table 1:** List of Inhibitors of mTOR Towards Breast Cancer

Sl. no	Inhibitors name	PubChem ID	Ref
1	Temsirolimus, CCI-779	6918289	Chan et al., 2005
2	Everolimus,RAD001	6442177	Shavetaet al., 2013
3	Rapamycin, sirolimus	5284616	Jerusalem et al., (2014)
4	Ridaforolimus, deforolimus,	11520894	HareHarvey et al., 2017
5	AZD2014	25262792	Guichard et al., (2015)
6	AZD8055	25262965	Jordan et al., (2014)
7	INK 128, MLN0128	45375953	Gokmen-Polar et al., (2012)
8	CC-223	58298316	Bendell et al., (2015)
9	Palomid 529 (P529),	11998575	Xiang et al., (2011)
10	OSI-027	44224160	Bhagwat et al., (2011)
11	Torin 1	49836027	Hall et al., (2012)
12	PP242	25243800	SparksGuertin (2010)
13	PP30	24905154	Leung et al., (2015)
14	KU-0063794	16736978	Lee et al., (2013)
15	XL388	59604787	Liu et al., (2009)
16	WYE-125132(WYE-132),	25260757	Chen et al., (2016); Yu et al., 2010
17	WYE-687	25229450	Yu et al., (2009)
18	WAY-600	25229526	Yu et al., (2009)
19	WYE-354	44219749	Yu et al., (2009)
20	Torin 2	51358113	Liu et al., (2013)
22	GDC-0349	59239165	Pei et al., (2013)
23	NVP-BEZ235, BEZ235	11977753	Serra et al., (2008)
24	PF-04691502	25033539	Wander et al., 2013
25	NVP-BGT226	57336745	Markman et al., (2012)
26	Apitolisib,GDC-0980	25254071	Wallin et al., (2011)
27	PF-05212384, PKI-587	44516953	Fouqué et al., 2016
28	SAR245409 (XL765)	49867926	Papadopoulos et al. (2014)
29	GSK2126458 (Omipalisib)	25167777	Munster et al., 2015
30	PKI-402	44187953	(Mallon et al., (2010))
31	PI-103	9884685	Jang et al., 2015
32	Metformin	4091	Zakikhani et al., 2007
33	Antrocin (AKT/mtor)	53474706	Rao et al., 2010
34	Piperlongumine	637858	Shrivastava et al., 2014
35	VS-5584 (SB2343)	46912230	Kolev et al., 2014
36	PKI-179	46947264	Venkatesan et al., 2010
37	Resveratrol		He et al., 2011
38	Osthole	10228	Hung et al., 2011
39	SF1126	66577114	Mahadevan et al., 2012
40	MKC-1	5327686	Schneider et al., 2008

**Table 2:** Selected Final Compounds from Re-Docking of Knowninhibitors Into the Active Site of the Protein Kinase for Virtual Screening

Name of Inhibitors	Docking Score (kcal mol-1)	Glide E model kJ/mol	Glide Energy (kcal/ mol)	Interactive residues	π- π Interaction Yes/No
sf1126	-8.705	-85.06	-74.137	Asp641, Cys679, Ala682, Leu683, Lys690, Asn725	No
WYE-687	-7.692	-82.145	-61.005	Ser711, Arg718	No
PKI-587	-7.617	-88.447	-45.599	Leu739	No
NVP-BGT226	-7.284	-64.903	-49.687	Glu701	Yes
KU-0063794	-7.174	-67.057	-47.904	Ala666, Ser668	No
GDC-0980	-7.036	-82.006	-59.175	Ser711, Ser719	No
WYE-354	-6.97	-82.607	-56.887	Arg718	No
Torin 2	-6.839	-48.904	-44.086	Ser738	No
XL388	-6.826	-77.967	-50.654	-	No
GDC-0980	-6.719	-78.072	-57.72	Ser719	No

**Table 3:** Structure Based Virtual Screening Results

S. No.	Compound ID	Docking Score (kcal/ mol)	Glide E model kJ/mol	Glide Energy (kcal/ mol)	Interactive residues for H-bond between IN-Ligand	π- π Interaction Yes/No
1	ZINC85569445	-10.607	-82.947	-61.06	Glu662, Lys690, Glu701, Asn725	Yes
2	ZINC14640443	-10.437	-52.385	-43.829	Ala682, Leu683, Lys690, Ser719	No
3	ZINC85489178	-10.434	-72.949	-48.322	Glu662, Arg716, Ser719, Ser722, Thr731	No
4	ZINC18208633	-10.429	-58.417	-49.658	Glu701, Pro715	No
5	ZINC85569455	-10.391	-83.908	-57.087	Glu662, Lys690, Glu701	Yes
6	ZINC85569435	-10.352	-76.75	-58.258	Glu662, Asn691, Glu701, Thr731	Yes
7	ZINC06446612	-10.144	-102.15	-63.041	Glu662, Ser719	Yes
8	ZINC08694341	-9.996	-82.165	-61.169	Glu662, Ser719	No
9	ZINC85569217	-9.921	-65.376	-45.512	Thr731	Yes
10	ZINC08791845	-9.869	-82.457	-52.494	Glu662	No

**Figure 2:**
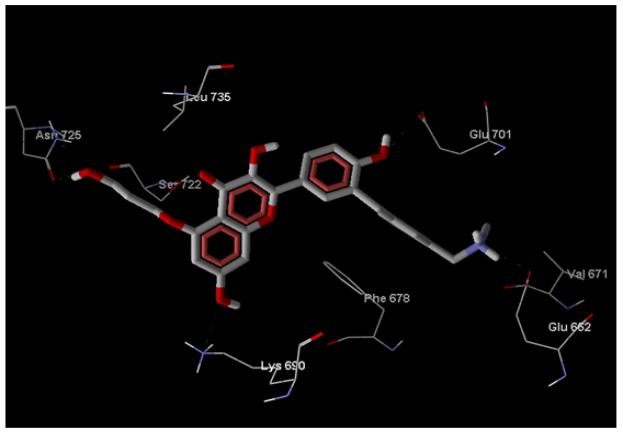
Hydrogen Bond Interaction betweenCompound ID: ZINC85569445 and the Target Protein mTOR

**Figure 3:**
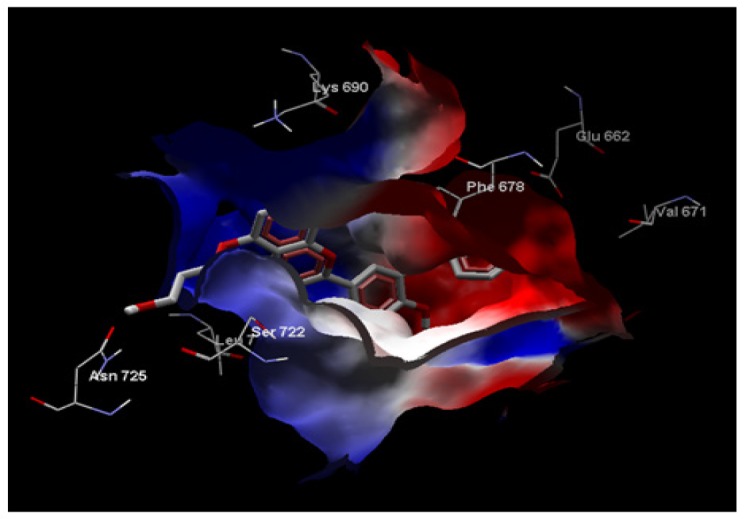
Electrostatic Interaction between the Compounds ID: ZINC85569445 in the Active Site of mTOR

**Table 4:** ADME Profile of Screened Hits

S. No.	Compound ID	MW	CNS	HBD	HBA	QPlogPo/w	QPlogS	QPlogBB	QPlogKP	Rule of 5	Rule of 3
1	ZINC85569445	477.513	-2	5	8.7	2.392	-4.191	-2.343	-5.648	0	2
2	ZINC14640443	288.256	-2	3	3.75	1.399	-2.801	-2.351	-4.4	0	0
3	ZINC85489178	470.694	-2	5	5.25	2.849	-5.239	-2.617	-7.405	0	2
4	ZINC18208633	388.425	0	3	4	3.328	-3.894	-0.435	-4.639	0	0
5	ZINC85569455	475.54	-2	4	6	3.703	-5.389	-1.943	-5.472	0	1
6	ZINC85569435	463.486	-2	5	8.7	2.099	-3.808	-2.116	-5.593	0	2
7	ZINC06446612	495.58	-2	1.25	7.75	4.857	-5.732	-1.339	-0.536	0	1
8	ZINC08694341	477.562	-2	1.25	8.5	4.211	-5.722	-1.462	-1.189	0	1
9	ZINC85569217	476.528	-2	5	3.75	4.397	-6.134	-2.095	-3.315	0	2
10	ZINC08791845	439.473	-1	2	7	4.402	-6.317	-0.868	-1.073	0	1

MW (Molecular Weight of the molecule), (130.0 to 725.0); CNS, predicted central nervous system activity on a -2 (inactive) to +2 (active) scale; HBD, Hydrogen Bond Donor = (0.0 to 6.0); HBA, Hydrogen Bond Acceptor = (2.0 to 20.0); QPlogP o/w (Predicted octanol/water partition coefficient) = (-2.0 to 6.5); QPlogS, (Predicted aqueous solubility, logS) = (-6.5 to 0.5); QPlogBB, (Predicted brain/blood partition coefficient) = (-3.0 to 1.2); QPlogKP, (Predicted skin permeability, logKp) = (-8.0 to -1.0); Rule of 5, (Number of violations of Lipinski’s rule of five) = (maximum is 4); Rule of 3, Violations (Number of violations of Jorgensen’s rule of three) = (maximum is 3)

**Figure 4:**
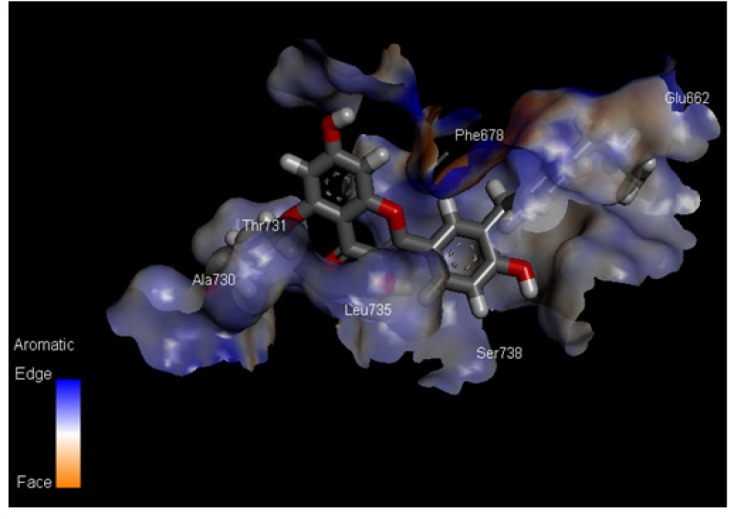
Aromatic Interactions between the most Effective Compound ID: ZINC85569445 and mTOR Protein

**Figure 5:**
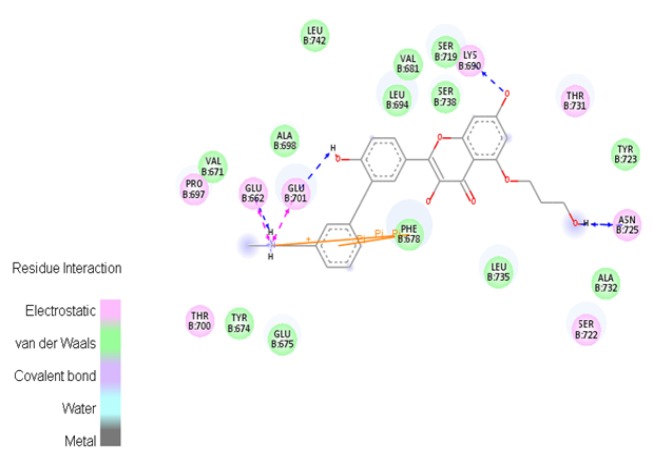
Interactions of the Compound ID: ZINC85569445 in the Active Site of mTOR

**Figure 6:**
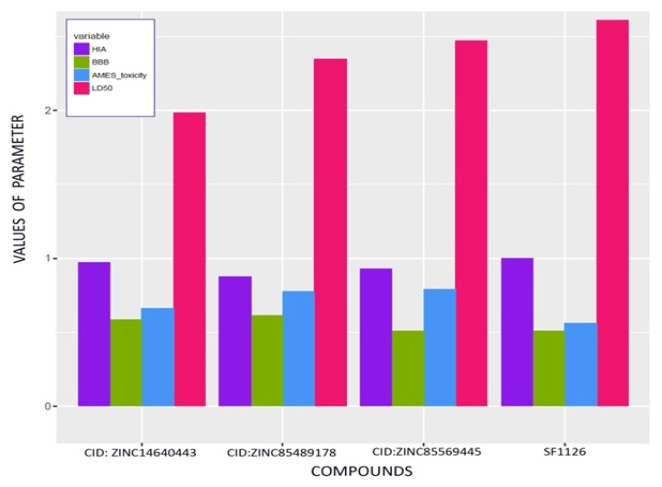
Comparative HIA, BBB, and LD50 of the Established Compound against Virtual Screened Compounds

**Figure 7:**
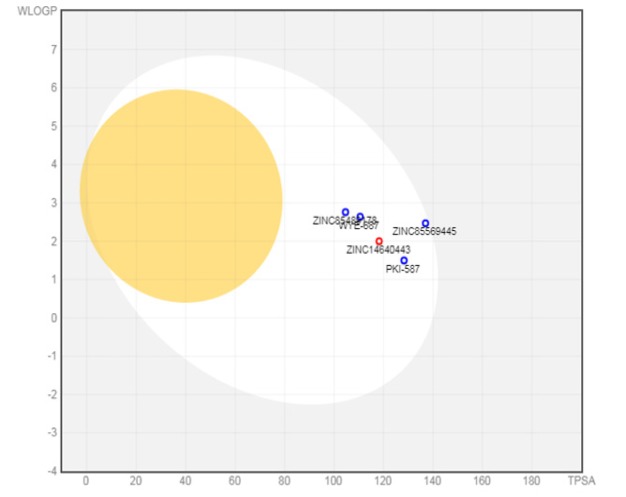
Boiled Egg Plot Predicted for the Best 3 Compounds from Established Drugs and Virtual Screened Drugs

**Table 5:** Comparative ADMET Profile of Best Established Dock Compound and Test Ligands

Compound	HIA	BBB	CYP substrate/inhibition	AMES toxicity	Carcinogenicity	LD50 in rat
SF1126	0.9992	0.5096	Non-substrate/non-inhibitor	0.5621	Non-carcinogen	2.6105
ZINC85569445	0.9298	0.5101	Non-substrate/non-inhibitor	0.7878	Non-carcinogen	2.4722
ZINC14640443	0.9704	0.586	Non-substrate/inhibitor	0.663	Non-carcinogen	1.9822
ZINC85489178	0.8774	0.6136	Non-substrate/non-inhibitor	0.7778	Non-carcinogen	2.3465

**Table 6:** Binding Free Energy Analysis Results

S. No.	Compound ID/Name	ΔG_bind_^a^	ΔG_coulomb_^b^	ΔG_covalent_^d^	ΔG_vdW_^c^	ΔG _sol GB_^e^
1	SF1126	-36.926	-22.993	15.799	-54.637	55.852
2	ZINC85569445	-89.038	-65.12	9.296	-55.945	75.929
	ZINC14640443	-48.027	-29.584	9.153	-29.07	26.395
4	ZINC85489178	-90.039	-55.047	6.71	-41.326	66.435
5	ZINC18208633	-68.198	19.778	1.678	-47.657	-2.161
6	ZINC85569455	-84.579	-47.073	6.579	-49.289	64.133
7	ZINC85569435	-82.428	-61.031	7.617	-49.668	67.263
8	ZINC06446612	-85.144	14.521	2.577	-60.392	1.39
9	ZINC08694341	-81.948	10.026	10.091	-58.752	7.041
10	ZINC85569217	-69.215	-12.936	10.077	-47.402	32.732
11	ZINC08791845	-73.241	13.789	11.497	-57.52	5.618

Energies in kcal mol-1; a, Free binding energy; b, Coulomb energy contribution to the binding free energy; c, Covalent energy contribution to the binding free energy; d, Van der Waals energy contribution to the binding free energy; e, The generalized born electrostatic solvation energy contribution to the binding free energy.

**Table 7:** Best Three Compound from -Established Dock Result and Virtual-Screened Dock Result Used for BOILED-Egg Plot

Molecule	MW	TPSA	MLOGP	GI absorption	BBB permeant
SF1126	852.84	344.2	-6.42	Low	No
WYE-687	528.61	110.53	1.61	High	No
PKI-587	615.73	128.29	1.21	High	No
ZINC85569445	478.51	136.97	-2.84	High	No
ZINC14640443	288.25	118.22	0.48	High	No
ZINC85489178	471.7	104.61	-3.51	High	No

*Hydrogen Bond interaction between compound ID and target protein*

ZINC85569445 and the target protein mTOR is shown (Figure 2). Green dotted lines represent the Hydrogen interaction between atoms. This interaction involving atoms of the residues Asn725, Glu701, Val67, Ser722, Lys690 of mTOR.

*Electrostatic Interaction between the compounds ID*

ZINC85569445 in the active site of mTOR shown in (Figure 3). The red surface of the protein is electrically negative surface;while, the blue surface is electrically positive. The compound is deeply embedded in the cavity of positive and negative amino acids of the target protein mTOR.

*Aromatic interactions between the most effective compoundID*

ZINC85569445 and mTOR protein shown in (Figure 4). Most favorable region of the aromatic interaction is the blue region (Edge), where Phe678 of mTOR is actively participating in bond formation. While the least favorable region (Face) have less interaction.

*Interactions of the compounds ID*

ZINC85569445 in the active site of mTOR shown in [Fig 5]. The residues (Asn725, Glu701, Val67, Ser722, and Lys690) of blue dotted line participating in Hydrogen bond interaction while the residues in green circles forming Van-der Waals. Pink circled residues participating in electrostatic interaction.

*ADME profile*

An ADME property of the top 10 hits was calculated by evaluating their physicochemical properties using QikProp, Schrodinger. All the predicted ADME properties are accepted within the standardized range defined for human use such as Molecular weight (MW 130-500), H-Bond donor (< 5.0), H-Bond acceptors (< 10.0), the octanol/water partition coefficient log (-2 to 6.2), the aqueous solubility log (mol/L) (-6.5 to 0.5), CNS activity -2 (inactive) to +2 (active Lipinski’s rule of five and Jorgensen rule of three), shown in Table 4.

*Comparative ADMET profile of the test ligands and the control*

Comparative studies of the parameters such as Human intestinal absorption(HIA), Blood-brain barrier (BBB), AMES toxicity, LD50 doses of best established docked compound SF1126 (control) and top three virtual screened ligands (CID: ZINC85569445, CID: ZINC14640443, and CID: ZINC85489178) was done: using R-programming language which is depicted in Figure 6. ADMET parameters such as HIA, BBB, AMES toxicity, and LD50 are predicted using online web server tool admetSAR server shown (Table 5). Derived properties revealed that CID: ZINC85569445, CID: ZINC14640443 had the better blood-brain barrier which was higher than the control molecule SF1126. SF1126, CID: ZINC85569445 showed almost equal HIA probability higher than others. Higher HIA denotes the compound could be better absorbed from the intestinal tract after oral administration. Computed result for LD50 dose showed best for SF1126. ZINC85569445 had lower LD50 value compare to another compound. CID: ZINC85489178 had almost equivalent LD50 dose to SF1126. Regarding toxicity, the probability of being toxic was lower for SF1126 and ZINC85569445. Ames test employed to test whether a compound is toxic or not. The result of the Ames test revealed that all ligands and control molecule were non toxic.

*MM-GBSA*

The binding free energy was calculated using a post-scoring method –MMGBSA for the evaluation of molecular docking process. Obtained MMGBSA (ΔG bind) ranges from -58.149 to -89.989, are presented in Table 4. Results were correlated along with docking score to design a relevant drug like potent inhibitors. Higher binding free energy of the lead defines the greater affinity to bind with the receptor.

*BOILED-Egg plot analysis*

To understand the poor pharmacokinetics behaviors and bioavailability of the drug which is crucial for drug discovery process, BOILED-Egg plot is proposed as an accurate model to predict lipophilicity and polarity of the small molecule. Prediction for both BBB and intestinal permeation is translated into a molecular design on account of speed, reliability, conceptual clarity and clear graphical output of the model (Table 5) indicates the pharmacokinetics and bioavailability properties of the best six compound; three from established docking and three from virtual screening. It suggests that there is low GI absorption for best pre-established docked compound while high GI absorption for the virtual screening compound. The result itself indicates that there is no BBB permeant. All the virtual screened drugs are in white space meaning proper GI-intestinal absorption. BOILED-Egg plot is shown (Figure 7).

## Discussion

Breast Cancer is considered as the prime cause of mortality among women worldwide. Metastatic breast cancer is a diverse disease with different subtypes and irremediable with current treatment regimens (Brouckaert et al., 2017). Based on the data composed by American cancer society, nearly 231,840 recent cases supposed to be diagnosed in 2105 (Ward et al., 2015). One important cause of cancer is the sequential mutation in the number of genes due to the fact of genetic instability and environmental factors (Al-Hajj et al., 2003). mTOR (mammalian target of rapamycin) is a serine/threonine kinase, which controls the various acts of our body such as cell growth, survival, metabolism, is upregulated in various cancer. mTOR is a downstream member of the PI3K/AKT signaling pathway, and its aberrant activation is observed in various human malignant diseases (Vicier et al., 2014). Therefore, mTOR is considered as a desirable target for treatment strategies.

Accordingly, substantial importance is given on PI3K/AKT/mTOR signaling pathway which has led to the progress of different kinds of inhibitors. In the present study, we found a pre-existing drug against mTOR and technically found SF1126 with the best binding score -8.705kcal/mol against mTOR. Further exploration established from the structure similarity search of SF1126 against ZINC database found various compound with similar properties against mTOR. High-throughput virtual screening performed in this study for structure similarity search which revealed ZINC85569445 with best binding score -10.607kcal/mol. In this study the tested compound CID: ZINC14640443 displayed with the lowest binding energy of -10.607kcal/mol. The binding energy of the control molecule SF1126 was higher than CID: ZINC14640443 as found in our study, thus CID: ZINC14640443 displayed the much better binding score. ADME profile of both the compounds SF1126(control) and test ligand ZINC85569445 was evaluated using QikProp Schrodinger and all the parameters accepted within the standardized range defined for human use. CID: ZINC14640443 predicted blood-brain barrier 0.51 compare to SF1126 of 0.50. Carcinogenic profile of both the test ligand and control molecule revealed to be non-carcinogenic.

The effectiveness of the known inhibitors against mTOR has been tested with significant antiproliferative activity while some of them are being under phase II/III clinical trials (Chresta et al., 2010). In spite of having high ability in inhibiting the activity of mTOR, inhibitors are quite inadequate in fighting against cancer. The likelihood, there is the number of reasons such as feedback loop can activate the upstream signaling pathway and promote cell survival and proliferation. The mTOR signaling pathway is essential for healthy cell growth and viability and its inhibition can be destructive to other cells and tissues(Xie et al., 2016). Despite some loopholes, further attention should be given to the understanding of the mTOR signaling pathway and its downstream processing which play a vital role in tumor progression.

In conclusion, this research study focuses on finding a satisfactory lead compound which targets to inhibit the breast cancer. Phosphatidylinositol 3- kinase/AKT/ mammalian target of rapamycin(PI3K/AKT/mTOR) pathway is dysregulated in various cancer along with the breast cancer and is the major signaling pathway which controls the routine activity of our body including cell survival, cell division, etc. In this study, we found many pre-existing compounds against the mTOR protein and technically found SF1126 with the high-affinity properties along with the best binding energy score. Further research progress based on the structural similarity of SF1126 against drug database found that there are several more compound having property against mTOR. Best drug found in this research is ZINC85569445and concluded based on the ADME profile and BOILED-Egg plot prediction. 

BOILED -Egg plot is a good informant for lead optimization and following analyses the virtual screened compound ZINC85569445 exhibits interesting pharmacokinetics. Virtual tested drugs are correctly placed in the white region of the egg which indicates that it constitutes preferable compound against mTOR protein. The binding free energy using post scoring method MMGBSA calculated for the evaluation of molecular docking, also concluded the virtual screened compound was having higher affinity with the receptor as compared to pre-exist drug. 

Conclusively the lead compound is ZINC85569445 ideal for the study of pharmacophore profile. The compound shows the best affinity among all the pre-exit drugs, and inhibition property against the target mTOR as well as the study of pharmacophore mapping of the compound displayed considerable binding affinity in the active site of protein mTOR. This research encourages suitable opportunity for compound ZINC85569445 to prevent breast cancer.

## Ethics approval and consent to participate

Not applicable.

## Competing interests

The authors declare that they have no competing interests.
